# Mapping interaction quality for nursing and medical students in primary care placement in municipal emergency care units: a systematic observational study

**DOI:** 10.3389/fmed.2024.1181478

**Published:** 2024-01-22

**Authors:** Solveig Giske, Siv M. Gamlem, Marit Kvangarsnes, Bodil J. Landstad, Torstein Hole, Berit Misund Dahl

**Affiliations:** ^1^Department of Health Sciences in Ålesund, NTNU - Norwegian University of Science and Technology, Ålesund, Norway; ^2^Department of Pedagogy, Volda University College, Volda, Norway; ^3^Ålesund Hospital, Møre and Romsdal Hospital Trust, Ålesund, Norway; ^4^Faculty of Health Sciences, Mid Sweden University, Östersund, Sweden; ^5^Unit of Research, Education and Development, Östersund Hospital, Östersund, Sweden; ^6^Medical Department, Ålesund Hospital, Møre and Romsdal Hospital Trust, Ålesund, Norway; ^7^Faculty of Medicine and Health Sciences, NTNU - Norwegian University of Science and Technology, Trondheim, Norway; ^8^Department of Public Health, University of Stavanger, Stavanger, Norway

**Keywords:** classroom assessment scoring system, clinical placement, interaction quality, medical students, nursing students, primary healthcare, student placement, systematic observations

## Abstract

**Introduction:**

Primary care placement for nursing and medical students is vital for developing the competence to accommodate the increasing number of patients with multimorbid and complex conditions. Prior studies have suggested that interaction quality in primary care placement empowers learning. However, research mapping interaction quality in primary care placements in municipal emergency care units is lacking. This study aimed to systematically map interaction quality for nursing and medical students in primary care placement in two municipal emergency care units.

**Materials and methods:**

This study adopted a systematic descriptive observational design. Systematic observations (*n* = 201 cycles) of eight nursing students (*n* = 103 cycles) and six medical students (*n* = 98 cycles) were used to map interaction quality across six learning situations between March and May 2019. Observations were coded using the Classroom Assessment Scoring System-Secondary (CLASS-S). Data were analyzed using descriptive statistics and Spearman correlations.

**Results:**

Interaction quality is described in three domains: (I) *emotional support,* (II) *framework for learning,* and (III) *instructional support*, and the overall measure, *student engagement*. The results indicated middle-quality interactions in the *emotional* and *instructional support* domains and high quality in the *framework for learning* domain and *student engagement.* Correlations exhibited similar patterns and ranged from non-significant to strong correlations.

**Conclusion:**

The interaction qualities indicated a generally positive and supportive learning environment contributing to nursing and medical students’ learning and active participation in work tasks related to their professional roles. Thus, this new form for primary care placement for nursing and medical students in the municipal emergency care units was found to be a positive learning arena. These results may enhance nursing and medical education programs in countries with similar health services and education. Health education, supervisors, peers, and others contributing to students’ learning should recognize which interaction qualities may affect learning and how to improve quality, thus affecting supervisors’ approach to training students. While the CLASS-S showed potential for mapping interaction qualities for nursing and medical students in primary care placement in municipal emergency care units, further studies are needed to validate the CLASS-S for use in clinical placement settings.

## Introduction

Learning in clinical placement is considered vital for nursing and medical students to integrate theoretical knowledge and practical skills and form a professional identity for becoming a competent nurse or physician (1, 2). Nursing and medical education have been criticized for not adapting to societal changes involving the growing number of patients with multimorbidity and complex conditions and the transfer of healthcare services from secondary to primary healthcare. These changes have precipitated the need to develop students’ competence through primary care placements to meet the primary healthcare system’s patient care and treatment demands (3, 4). Norwegian municipalities offer emergency inpatient care for patients with somatic, substance use, and mental health challenges in municipal emergency care units^1^ (5). Studies have demonstrated that these units require nurses with advanced clinical competence and physicians with general medical expertise and skilled in leading complex interprofessional collaborations (6–8). Undergoing clinical placement in municipal emergency care units may contribute to preparing nursing and medical students for future work in this area. Furthermore, studies have reported that vital aspects of nursing and medical students’ learning process in primary care placement occur in interactions with supervisors, peers, interprofessional collaborations and patients (9–11). However, the quality of those interactions may influence students’ learning experiences (12, 13). Interaction quality in primary care placement in municipal emergency care units for nursing and medical students has not been studied previously. Therefore, this study aimed to map interaction quality for nursing and medical students in primary care placement in two municipal emergency care units.

Prior research has indicated that nursing and medical students in primary care placements value quality interactions that contribute to an engaging and inclusive learning environment, wherein supervisors facilitate a gradual progression in learning activities in their respective community of practice (CoP) (9, 14, 15). Further, supervisors who demonstrate an interest in students’ learning by being prepared and engaged reportedly contribute to a safe learning environment that facilitates active participation (16, 17). These results are supported by Bos et al. (18), who reported that nursing students’ motivation for learning is strongly associated with supervisory relationships and a pedagogical atmosphere. Moreover, Thyness et al. (19) found that medical students were more likely to observe rather than actively participate in learning activities when they felt unsafe.

Nursing and medical students have reported that proximity to one or two supervisors contributes to a trusting supervisor relationship that supports students’ confidence in asking questions and showing professional weakness (20, 21). Nursing and medical students have also suggested that supervisor proximity helps supervisors more accurately assess students’ knowledge and skills and assign appropriate work tasks related to their learning goals (13, 22). Additionally, proximity contributes to supervisor feedback that is timely, constructive, and adapted to students’ levels of competence and clinical performance (13, 20). Interestingly, Bates et al. (23) found that medical students interpreted critical feedback as supportive of learning—rather than challenging—when the supervisor relationship was built on trust.

Reportedly, a trustful supervisor relationship promotes opportunities for professional and ethical discussions that may further stimulate learning and encourage self-confidence and professionalism (12, 21). Studies have indicated that nursing and medical students value supervisors who ask questions, take time to listen to students’ thoughts, and show interest in their knowledge and perceptions (24, 25). Nursing students report feeling invisible, worthless, and that they are missing out on discussion opportunities when supervisors fail to engage in professional discussions (26). Further, studies have demonstrated that nursing and medical students consider their supervisors important professional role models in terms of how they interact with patients, peers, and the students themselves, thus contributing to students’ professional development (18, 21, 27). In Salminen et al. (13) study, medical students reported that supervisors who were friendly to patients were also friendly to students.

While the aforementioned literature focuses on supervisor–student interactions, nursing and medical students have also been found to value learning through interacting interprofessionally and with patients (28, 29). Studies have demonstrated that interprofessional collaboration enables students to learn about each other’s professional roles, view patients’ situations from a holistic perspective, and communicate with each other effectively and respectfully (29, 30). Additionally, professional and ethical reflections are shown to contribute to knowledge and interprofessional understanding (31).

Prior research has suggested that patients are generally optimistic about letting students practice on them (32, 33). Moreover, patients’ goodwill toward students is crucial to learning, and medical students have reported that patients who express a desire to contribute to their learning motivate the former to spend more time asking questions and conducting examinations (19).

While previous studies report on interaction qualities important for nursing and medical students’ learning in primary care placement, research that systematically maps it in placements in municipal emergency care units is lacking. Thus, this study aimed to systematically map the interaction quality for nursing and medical students in primary care placement in two municipal emergency care units and to answer the following research question:

What is the interaction quality for nursing and medical students in primary care placement in municipal emergency care units?

## Materials and methods

### Design

This study adopted a systematic descriptive observational research design^2^ (34, 35). To map interaction quality, systematic observations of nursing and medical students across six learning situations were conducted and coded using the Classroom Assessment Scoring System-Secondary (CLASS-S) (36).

### Participants and setting

Eight nursing students (out of 35 eligible), four from each of two Norwegian universities, and six medical students (out of 19 eligible) from one of those universities were conveniently sampled based on fulfilling the following inclusion criteria (34): (1) fourth-semester nursing students attending medical clinical placement at a local hospital or medical center, (2) ninth-semester medical students attending medical clinical placement at a local hospital, (3) Norwegian speaking, and (4) interested in participating. The municipal emergency care units’ management recommended the number of students based on academic and structural resources. The nursing students (seven females and one male) were aged 20–41 years (median age = 22 years). The medical students (four females and two males) were aged 24–28 years (median age = 26).

The municipal emergency care units were located in two cities in mid-Norway and were chosen based on professional affiliations with the two universities. The nursing and medical students participated in a new form for clinical placement where they trained on their professional role in collaboration with each other, supervisors, other colleagues, and patients. This placement lasted 2 weeks and replaced 2 weeks of their original medical clinical placement. The students were divided into four groups, each containing two nursing students and one or two medical students. The groups took turns completing their clinical placements. Two groups were placed in each municipal emergency care unit based on geographical proximity. Based on the units’ structure, a daily step-by-step plan was developed to facilitate six learning situations: morning meetings, preparations, pre-rounds, rounds, clinical work, and afternoon meetings. Additionally, the plan also included a schedule for the systematic observations (Table 1).

**Table 1:** The day’s plan, characteristics of the learning situations, and schedule for the systematic observations.

Step by step	Activity	Supervision
Morning meeting 08.00–08.30 a.m. *Observation 1* The researchers adjust the time for the observation.	The students obtain morning reports and work tasks for the day are distributed.	Physician and nurse mutual
Preparation 08.30–10.00 a.m. *Observation 2* The researchers adjust the time for the observation.	Medical students: They read about their patients (discharge or progress notes, medicine administration records, blood tests, drug coordination, interaction search, and so on.).	Physician
	Nursing students: They orient themselves to their patients, collect blood samples, and record vital signs to ensure that they are ready for pre-rounds. Their responsibilities include their patients’ care and food and performing any procedures; they provide medications in consultation with their supervisor	Nurse
Pre-rounds and rounds *Observations 3 and 4*	The students participate in the pre-rounds and rounds.	Learning in communities of practice
Clinical work *Observation 5* The researchers observe relevant learning situations	The students conduct clinical work in collaboration with their supervisors. They document their assigned patients’ daily progression or discharge notes. Other activities include: admitting patients (new patients who then become the students’ new patients) procedures (all patients) writing the note for supervision	Learning in communities of practice
Afternoon meeting At 2 p.m. *Observation 6*	One student presents a learning situation The note for supervision comprises the following: The situation The challenge, problem, and task Their own thoughts concerning solutions The student receives questions and knowledge from supervisors and students, which results in reflection and novel insights.	The action and reflection model

### Measure

The Classroom Assessment Scoring System-Secondary (CLASS-S) (36) is an observational tool designed to assess the quality of teacher–student interactions as a starting point for learning in secondary and upper secondary schools (36, 37). The CLASS-S has been validated and used for classroom research in Norway (38, 39), the USA (37, 40), and Finland (41). According to a meta-analysis of the CLASS factor structure, it represents the data effectively (42), thus verifying the CLASS-S′ efficacy as a tool for studying teacher–student interactions.

The CLASS-S measures interaction quality via three domains—namely, *emotional support, classroom organization,* and *instructional support,* and an overall measure of *student engagement* (36, 40). Furthermore, each domain has three–five dimensions (Table 2) that are based on developmental theory (44) and a sociocultural learning perspective (15, 45). Each dimension and the overall measure of *student engagement* contain custom behavioral markers forming the basis and guide the observer for scoring on a 7-point Likert scale, wherein 1–2 = low quality, 3–5 = middle quality, and 6–7 = high quality (36). However, *negative climate* is reversed, meaning low scores indicate rare or absent occasions of negativity (36, 38). The scores are registered on an observation form—the Secondary CLASS Score Sheet^3^ (43). Overall, the dimensions’ quality indicates the extent to which teacher–student interactions support learning (36). The CLASS-S manual suggests that the systematic observations are organized in 25-min cycles of 15–20 min of observation and note-taking, followed by 10 min of scoring (36, 39). However, observation cycles may be interrupted owing to unexpected circumstances, and hence, a minimum of 8 min of observational time is considered acceptable for scoring (36).

**Table 2:** Overview of the CLASS-S and descriptions of the dimensions and overall measure (36).

Domains	Dimensions	Descriptions
Emotional support	Positive climate	Reflects the emotional connection, relationships, and respect communicated among teachers and students.
	Teacher sensitivity	Reflects the teachers’ timely responses to the academic, social, emotional, behavioral, and developmental needs of individual students and the whole class.
	*Regard for adolescent perspective (omitted because the students are adults)*	*Reflects the degree to which teachers meet and capitalize on adolescents’ social and developmental needs and goals by facilitating autonomy and leadership, and the extent to which students’ ideas and opinions are valued, utilized, and made relevant.*
Framework for learning *(renamed from classroom organization because the setting was in a clinical environment)*	Behavior management	Reflects the teachers’ methods for encouraging desirable behaviors and preventing and redirecting misbehavior.
	Productivity	Captures how well teachers manage time and routines to ensure that instructional time is maximized and downtime is minimized.
	Negative climate	Reflects the overall negativity level among teachers and students, which includes aspects such as frequency, intensity, and quality (e.g., irritability, yelling, and humiliation).
Instructional support	Instructional learning formats	Focuses on how teachers maximize student engagement by facilitating learning through a clear presentation of the learning objectives, materials, and interesting activities.
	Content understanding	Refers to the depth of the lessons and approaches used to help students grasp the framework, central ideas, and procedures within their academic discipline.
	Analysis and inquiry	Reflects the degree to which students are engaged in higher-order thinking to solve problems, tasks, and questions by utilizing their knowledge, skills, and metacognition.
	Quality of feedback	Refers to how teachers’ and peers’ feedback expands and extends students’ learning, understanding, and encourages their participation.
	Instructional dialogue	Captures teachers’ use of cumulative strategies that facilitate content-focused discussions aimed at fostering active student participating for them to achieve a deeper understanding of the content.
Student engagement		Captures the degree of the overall level of student engagement and functioning level in the classroom.

CLASS-S, Classroom Assessment Scoring System-Secondary.

The *emotional support* domain highlights that students’ social and emotional functioning in the classroom contributes to school success (36, 40). This domain is theoretically based on attachment (46) and self-determination theories, which illuminate an individual’s need for belonging and autonomy (47, 48), and contains three dimensions: *positive climate, teacher sensitivity,* and *regard for adolescent perspectives*. The domain *classroom organization* assumes that classrooms are well organized, with teachers managing students’ behavior, time, and tasks to provide the most learning opportunities (36). This domain is founded on theories of self-regulatory skills related to students’ cognition, efforts to achieve learning goals (49), and how learning occurs in social interactions in CoPs (15). This domain encompasses three dimensions: *behavior management, productivity,* and *negative climate.* The *instructional support* domain highlights the difference between merely learning facts and achieving a deeper understanding, with the teacher using strategies to facilitate learning (50). This domain builds on students’ cognitive and language development, which involves how they construct and develop knowledge in a meaningful way (51), and contains five dimensions: *instructional learning formats, content understanding, analysis and inquiry, quality of feedback,* and *instructional dialogue.* The overall measure for *student engagement* captures students’ overall engagement levels and functioning in the classroom (36).

To the best of our knowledge, the CLASS-S has not been used to systematically map the quality of teacher–student interactions for nursing or medical students in clinical placement settings. Nor did we find studies that systematically map interaction quality in clinical placement for nursing or medical students. The CLASS-S intends to examine the quality of teacher–student interactions in the classroom; nevertheless, we used it to study the overall quality of nursing and medical students’ interactions in primary care placement in two municipal emergency care units. The measure was adapted to fit the study setting; the domain *classroom organization* was renamed *framework for learning* because the setting was in a clinical environment and not a classroom. The dimension *regard for adolescent perspective* was omitted because the participants were adult students (Table 2).

### Data collection

Systematic observations were conducted between March and May 2019 by four of the six authors. Two and two researchers were present at the municipal emergency care units—taking turns observing a nursing or medical student across the six learning situations while interacting within the CoPs. Overall, the nursing and medical students were systematically observed 2 days per week during their two-week clinical placement. The systematic observations were coded according to the CLASS-S manual (36) and scored on individual Secondary CLASS Score Sheets^4^ (43).

We conducted 215 systematic observation cycles distributed among the nursing (*n* = 113 cycles) and medical students (*n* = 102 cycles). The observational time within the collected cycles ranged from 2 to 71 and 3 to 43 min for the nursing and medical students, respectively. Notably, 23 cycles exhibited <8 min of observational time, 8 of which exhibited <6 min of observation time; hence, we decided to include all cycles with a minimum observational time of 6 min. Moreover, cycles with missing data <5% were deleted listwise. After data cleaning, the number of systematic observations for analysis was 201 cycles distributed among the nursing (*n* = 103 cycles) and medical students (*n* = 98 cycles). The observational time within the collected cycles ranged from 6 to 71 and 6 to 43 min for the nursing and medical students, respectively.

Two pre-training sessions were carried out to strengthen interrater reliability, meaning the extent to which the data collectors agree and assign scores within one point to the same variable calibrated toward a master coder (36, 52). The second author, who is a certified user of the CLASS-S and thus considered a master coder, led the pre-training sessions and was responsible for the scoring in this study. The pre-training sessions resulted in calibrated scores and a coding consensus of more than 80%, which aligns with the CLASS-S manual (36). To further enhance interrater reliability during data collection, the researchers conducted daily meetings to calibrate the scores by discussing and justifying their coding.

### Data analysis

Data were analyzed using IBM SPSS version 28. Based on our data being ordinal, descriptive statistics of the median, mode, minimum (Min), and maximum (Max) values used to map interaction quality across the six learning situations. In addition, we have reported the mean to facilitate comparisons with previous and any future studies. We calculated the values for the domains and the dimensions. For the dimensions, a total score was calculated based on averages for the cycles overall and separately for the nursing and medical students (i.e., Positive climate (PC): PC1 + PC2 + PC3 + …. /103 = PC).

Bivariate correlations were assessed using Spearman’s rank order correlation as this is suitable for ordinal data. It measures monotonicity, whereas the more standard Pearson correlation coefficient measures linear dependence and is more appropriate for continuous variables (53). Two-sided *p* < 0.05 was considered statistically significant; we did not correct for *p*-values for multiple testing. We classified the correlation coefficient as follows: 0.1–0.29 (weak correlation); 0.30–0.49 (medium correlation); 0.50 or higher (strong correlation) (54).

Cronbach’s alpha and mean inter-item correlation values were calculated to determine the internal consistency reliability of the CLASS-S domains and overall for the dimensions and student engagement (Table 3) (54). The alpha values for the *emotional support* domain ranged between 0.34 and 0.64, which means low internal consistency. The mean inter-item values ranged between 0.21 and 0.48, which indicated acceptable internal consistency. The *framework for learning* domain showed alpha values ranging between 0.45 and 0.72, which meant that internal consistency ranged between low to acceptable values. The mean inter-item values ranged between 0.19 and 0.51, which indicated acceptable internal consistency. The domain *instructional support* showed strong internal consistency with alpha values between 0.82 and 0.84 and mean inter-item values between 0.50 and 0.53. For all dimensions and overall measure *student engagement,* the alpha values showed strong internal consistency with values of 0.87 both overall and separately for the nursing and medical students and acceptable mean inter-item values ranging between 0.37 and 0.39.

**Table 3:** Cronbach’s alpha and mean inter-item correlations for the CLASS-S domains, dimensions, and overall measure for student engagement, for all students and the nursing and medical students separately.

	Both professions together (*n* = 201)		Nursing students (*n* = 103)		Medical students (*n* = 98)	
	Cronbach’s alpha	Mean inter-item	Cronbach’s alpha	Mean inter-item	Cronbach’s alpha	Mean inter-item
Domains						
Emotional support	0.54	0.37	0.64	0.49	0.34	0.21
Framework for learning	0.60	0.40	0.45	0.19	0.72	0.51
Instructional support	0.83	0.52	0.84	0.53	0.82	0.50
Dimensions and overall measure	0.87	0.38	0.87	0.37	0.87	0.39

### Ethical considerations

The Norwegian Centre for Research Data (reference number 602973) approved this study. The students received oral and written information regarding the study. Following the ethical principles of the World Medical Association Declaration of Helsinki, we informed them that participation was voluntary, data would be kept confidential, and they could withdraw from the study at any time without justification or facing any adverse consequences (55). The students’ supervisors were orally informed about the study and verbally agreed to be observed while interacting with the students. The students obtained oral patient consent before the researchers observed interactions involving patients.

## Results

Interaction quality across students’ learning situations is described through the CLASS-S domains, dimensions, and overall measure of *student engagement*.

### Characteristics and distribution of the learning situations

Table 1 presents the characteristics of the learning situations. Table 4 presents an overview of the distribution of the students’ learning situations (*n* = 201 cycles) and those for the nursing (*n* = 103 cycles) and medical students (*n* = 98 cycles). The most frequently observed learning situation for all students and for the nursing and medical students separately was *clinical work*. The least observed learning situation for all students and the medical students was *the morning meeting*, whereas that for the nursing students was *rounds*.

**Table 4:** Overview of the distribution of the systematically observed learning situations for the nursing and medical students.

	Morning meeting (%)	Preparation (%)	Pre-round (%)	Round (%)	Clinical work (%)	Afternoon meeting (%)
Nursing and medical students (*n* = 201)	22 (10.95%)	39 (19.40%)	36 (17.91%)	23 (11.44%)	54 (26.87%)	27 (13.43%)
Nursing students (*n* = 103)	11 (10.95%)	21 (20.39%)	16 (15.53%)	10 (09.71%)	30 (29.13%)	15 (14.56%)
Medical students (*n* = 98)	11 (11.22%)	18 (18.37%)	20 (20.41%)	13 (13.27%)	24 (24.49%)	12 (12.00%)

### Interaction quality across learning situations overall

The overall results in the domains for the nursing and medical students presented median values in the upper-middle to high range (Table 5). The *emotional support* dimensions *positive climate* and *teacher sensitivity* exhibited median values in the upper-middle range. In the *framework for learning* domain, the dimensions of *behavior management* and *productivity* exhibited median values in the high range; *negative climate* (reversed scored) exhibited median values in the low range. The instructional support dimensions *instructional learning formats, content understanding, analysis and inquiry, quality of feedback,* and *instructional dialogue* exhibited median values in the upper-middle range; an exception here was *analysis and inquiry,* which exhibited a median value in the middle range. Finally, the overall measure of *student engagement* exhibited a median value in the high range.

**Table 5:** Descriptive statistics elucidating the CLASS-S domains, dimensions, and overall measure—for all students and the nursing and medical students separately.

	Both professions together (*n* = 201)					Nursing students (*n* = 103)					Medical students (*n* = 98)				
	Median	Mode	Min	Max	Mean	Median	Mode	Min	Max	Mean	Median	Mode	Min	Max	Mean
Domains															
Emotional support	6.00	6.00	2.50	7.00	5.73	6.00	6.00	2.50	7.00	5.70	6.00	6.00	3.50	7.00	5.77
Framework for learning	7.00	7.00	3.00	7.00	6.56	7.00	7.00	5.00	7.00	6.60	7.00	7.00	3.00	7.00	6.53
Instructional support	5.00	6.00	2.00	7.00	5.14	5.00	6.00	2.00	7.00	5.02	6.00	6.00	2.00	7.00	5.26
Dimensions															
Positive climate	6.00	6.00	3.00	7.00	5.60	6.00	6.00	3.00	7.00	5.58	6.00	6.00	4.00	7.00	5.62
Teacher sensitivity	6.00	6.00	2.00	7.00	5.86	6.00	6.00	2.00	7.00	5.82	6.00	6.00	3.00	7.00	5.91
Behavior management	7.00	7.00	3.00	7.00	6.52	7.00	7.00	4.00	7.00	6.55	7.00	7.00	3.00	7.00	6.48
Productivity	6.00	7.00	2.00	7.00	6.22	6.00	7.00	2.00	7.00	6.25	6.00	6.00	2.00	7.00	6.19
Negative climate†	1.00	1.00	1.00	4.00	1.05	1.00	1.00	1.00	2.00	1.01	1.00	1.00	1.00	4.00	1.09
Instructional learning formats	6.00	6.00	1.00	7.00	5.56	6.00	6.00	1.00	7.00	5.55	6.00	6.00	1.00	7.00	5.57
Content understanding	6.00	6.00	1.00	7.00	5.53	6.00	6.00	1.00	7.00	5.51	6.00	6.00	2.00	7.00	5.55
Analysis and inquiry	4.00	3.00	1.00	7.00	4.05	3.00	3.00	1.00	7.00	3.74	5.00	6.00	1.00	7.00	4.38
Quality of feedback	5.00	6.00	2.00	7.00	5.25	5.00	6.00	2.00	7.00	5.13	6.00	6.00	3.00	7.00	5.38
Instructional dialogue	6.00	6.00	1.00	7.00	5.28	5.00	6.00	1.00	7.00	5.17	6.00	6.00	1.00	7.00	5.40
Overall measure															
Student engagement	7.00	7.00	2.00	7.00	6.24	7.00	7.00	2.00	7.00	6.27	6.00	7.00	4.00	7.00	6.20

CLASS-S, Classroom Assessment Scoring System-Secondary; Min, minimum score; Max, maximum score. Likert scale: 1–2 = low range, 3–5 = mid-range, 6–7 = high range. †Scores for negative climate are reversed.

All the dimensions and the overall measure for *student engagement*—except *positive climate, behavior management,* and *negative climate* (reversed scored)—were scored within all three ranges. While the former two were scored between the middle and high ranges, the latter was scored between the low and middle ranges. All dimensions and the overall measure for *student engagement* received the highest possible score (Max = 7), except *negative climate* (Max = 4). The dimensions of *negative climate, instructional learning formats, content understanding, analysis and inquiry,* and *instructional dialogue* received the lowest possible score (Min = 1).

### Interaction quality across learning situations

The results for the nursing and medical students (separately) revealed that median values within the domains, dimensions, and overall measure for student engagement were reasonably similar (Table 5). In the dimensions, the most significant difference was found in the *analysis and inquiry* dimension, which exhibited a somewhat lower median value for nursing students than for medical students. Further, the minimum and maximum scores showed results that were the same as—or had a one-point difference in—the minimum scoring, except for *negative climate* and *student engagement*, which exhibited a two-point difference in the minimum or maximum scoring.

### Bivariate correlations among the CLASS-S dimensions and student engagement measure

For all the students, and the nursing and medical students separately (Tables 6–8, respectively), the two strongest correlations within the dimensions were between *instructional dialogue* and *quality of feedback* and between *content understanding* and *instructional learning formats.* The weakest correlation for all students was between *content understanding* and *negative climate*. The weakest correlation was between *analysis and inquiry* and *instructional learning formats* for nursing students and between *analysis and inquiry* and *teacher sensitivity* for medical students.

**Table 6:** Correlation matrix of scores for the quality of interactions for the nursing- and medical students.

CLASS-S dimensions and overall measure	PC	TS	BM	P	NC	ILF	CU	AI	QF	ID	SE
Positive climate	1	0.298**	0.076	0.288**	−0.1	0.255**	0.200**	0.127	0.275**	0.276**	0.384**
Teacher sensitivity		1	0.472**	0.536**	−0.216**	0.561**	0.497**	0.226**	0.429**	0.530**	0.577**
Behavior management			1	0.447**	−0.271**	0.420**	0.358**	0.135	0.322**	0.388**	0.387**
Productivity				1	−0.156*	0.531**	0.493**	0.262**	0.337**	0.413**	0.602**
Negative climate					1	−0.185**	−0.141*	0.044	−0.014	−0.154*	−0.242**
Instructional learning formats						1	0.617**	0.218**	0.493**	0.555**	0.494**
Content understanding							1	0.402**	0.513**	0.492**	0.420**
Analysis and inquiry								1	0.531**	0.477**	0.284**
Quality of feedback									1	0.690**	0.421**
Instructional dialogue										1	0.543**
Student engagement											1

**Correlation is significant at *p* < 0.01 (two-tailed). *Correlation is significant at *p* < 0.05 (two-tailed). *n* = 201 cycles. PC, Positive climate; TS, Teacher sensitivity; B, Behavior management; P, Productivity; NC, Negative climate; ILF, Instructional learning formats; CU, Content understanding; AI, Analysis and inquiry; QF, Quality of feedback; ID, Instructional dialogue; SE, Student engagement.

**Table 7:** Correlation matrix of scores for the quality of interactions for the nursing students.

CLASS-S dimensions and overall measure	PC	TS	BM	P	NC	ILF	CU	AI	QF	ID	SE
Positive climate	1	0.419**	0.19	0.321**	−0.077	0.288**	0.341**	0.094	0.259**	0.241*	0.425**
Teacher sensitivity		1	0.482**	0.533**	−0.147	0.580**	0.548**	0.267**	0.519**	0.507**	0.566**
Behavior management			1	0.418**	−0.113	0.427**	0.309**	0.175	0.354**	0.345**	0.347**
Productivity				1	−0.07	0.473**	0.463**	0.275**	0.337**	0.366**	0.584**
Negative climate					1	−0.135	−0.084	0.024	−0.031	−0.032	−0.072
Instructional learning formats						1	0.633**	0.195*	0.491**	0.586**	0.538**
Content understanding							1	0.423**	0.618**	0.561**	0.471**
Analysis and inquiry								1	0.519**	0.506**	0.295**
Quality of feedback									1	0.728**	0.436**
Instructional dialogue										1	0.524**
Student engagement											1

**Correlation is significant at *p* < 0.01 (two-tailed). *Correlation is significant at *p* < 0.05 (two-tailed). *n* = 103 cycles. PC, Positive climate; TS, Teacher sensitivity; B, Behavior management; P, Productivity; NC, Negative climate; ILF, Instructional learning formats; CU, Content understanding; AI, Analysis and inquiry; QF, Quality of feedback; ID, Instructional dialogue; SE, Student engagement.

**Table 8:** Correlation matrix of scores for the quality of interactions for the medical students.

CLASS-S dimensions and overall measure	PC	TS	BM	P	NC	ILF	CU	AI	QF	ID	SE
Positive climate	1	0.161	−0.037	0.250*	−0.126	0.231*	0.042	0.171	0.297**	0.305**	0.344**
Teacher sensitivity		1	0.456**	0.536**	−0.288**	0.544**	0.429**	0.199*	0.324**	0.570**	0.590**
Behavior management			1	0.475**	−0.368**	0.405**	0.414**	0.115	0.300**	0.440**	0.432**
Productivity				1	−0.207*	0.588**	0.518**	0.285**	0.348**	0.472**	0.613**
Negative climate					1	−0.231*	−0.187	0.012	−0.026	−0.244*	−0.343**
Instructional learning formats						1	0.595**	0.291**	0.519**	0.542**	0.450**
Content understanding							1	0.412**	0.390**	0.421**	0.365**
Analysis and inquiry								1	0.545**	0.417**	0.310**
Quality of feedback									1	0.639**	0.417**
Instructional dialogue										1	0.576**
Student engagement											1

**Correlation is significant at *p* < 0.01 (two-tailed). *Correlation is significant at *p* < 0.05 (two-tailed). *n* = 98 cycles. PC, Positive climate; TS, Teacher sensitivity; B, Behavior management; P, Productivity; NC, Negative climate; ILF, Instructional learning formats; CU, Content understanding; AI, Analysis and inquiry; QF, Quality of feedback; ID, Instructional dialogue; SE, Student engagement.

Generally, the correlations within the dimensions exhibited similar patterns, but some differences were observed when comparing nursing and medical students. While *negative climate* exhibited no significant correlations with any dimension for the nursing students, several weak significant negative correlations were noted for the medical students. For nursing students, the results exhibited weak significant correlations between *positive climate* and *teacher sensitivity*, and *positive climate* and *content understanding.* However, these dimensions did not exhibit significant correlations for the medical students.

Moreover, correlations involving the overall measure of *student engagement* revealed similar results for all the students and for the nursing and medical students separately. The strongest correlations were observed with *productivity* and *teacher sensitivity*, respectively. The weakest correlations were with *negative climate* for all students’ results (Table 6) and with *analysis and inquiry* for both nursing and medical students separately (Tables 7, 8, respectively).

## Discussion

This study aimed to map interaction quality for nursing and medical students in primary care placement in two municipal emergency care units. Based on the CLASS-S, systematic observations provided valuable information regarding the quality of interactions occurring while students participated in learning activities in the CoPs. The results revealed that the interaction quality was generally in the upper-middle range in the domains of *emotional support* and *instructional support* and high quality in *framework for learning*. We found a high degree of active student participation in the overall measure of *student engagement*. The correlations exhibited similar patterns for all students as well as for the nursing and medical students separately—ranging from non-significant to strong correlations.

In the *emotional support* domain, our results indicated that interaction quality was generally characterized by positive emotional relations and attention to students’ learning needs (36). Previous studies have demonstrated that nursing and medical students value quality interactions involving friendly and approachable supervisors, as such interactions promote a sense of belonging, trust, and safety (20, 21); otherwise, students have reported feeling ignored and unwanted, contributing to less favorable learning experiences (12, 56). Additionally, our results showed medium to strong positive correlations between the dimensions within the *emotional support* domain and the overall measure of *student engagement.* This indicates an important connection between emotional support and active student participation. These correlations concur with the studies of Lea et al. (16), O’Donoghue et al. (17), and Thyness et al. (19) suggesting that supervisors who show interest in students can improve student participation. Using the CLASS-S, we obtained information regarding the extent to which the interaction quality within the dimensions of the *emotional support* domain occurs in municipal emergency care units. The results provide useful information for these units to consider further development as a learning arena for nursing and medical students. Our results were in the upper-middle range, which suggests room for improvement. Supervisors and others participating in nursing and medical students’ primary care placement in municipal emergency care units should, therefore, recognize the importance of interactions that reflect a welcoming atmosphere, respectful communication, and attention to students’ learning needs.

Our results in the domain *framework for learning* exhibited high scores in the *behavior management* and *productivity* dimensions for all the students. According to the CLASS-S manual, high scores in these dimensions indicate that the students fulfilled behavioral expectations, were engaged, and participated actively in work tasks related to their professional roles (36). Previous studies have reported that clear expectations of work tasks and access to learning activities in CoPs contribute to active student participation, which further promotes learning on a peripheral trajectory (56, 57). By contrast, nursing and medical students have reported that being ignored or an absence of supervisor engagement in the CoP precipitates passivity and limited active participation (56, 57). In the CLASS-S manual, the degree of student participation in learning activities is examined through the overall measure of *student engagement* (36). Our results revealed medium positive correlations between *behavior management* and *student engagement* and strong positive correlations between *productivity* and *student engagement*, thus indicating a connection between active participation and student engagement.

For both nursing and medical students, we found generally low scores on the *negative climate* dimension, indicating rare or absent episodes of negativity. However, the results for the medical students exhibited a maximum score of four in one case, between the medical and nursing students themselves. Whelan et al. (58) demonstrated how negative interactions may affect medical students’ learning; they found that medical students’ experience of shame in clinical placement (related to supervisors humiliating them for lacking medical knowledge or treating them in a disrespectful way) reduces their confidence, engagement, and motivation. Additionally, Thyness et al. (19) found that medical students—when feeling unsafe—exhibit a learning style of passive observation rather than that of active participation. Similar findings have been reported in studies involving nursing students (12, 59). Despite our results indicating a low degree of negativity in the municipal emergency care units, supervisors and others contributing to nursing and medical students’ learning should avoid negative behaviors such as irritability, sarcasm, or disrespect, and be aware of how they may negatively affect student participation and learning. Based on our results, the CLASS-S seems useful for mapping the extent to which students’ active participation, related to the *framework for learning* domain, is facilitated in the municipal emergency care units.

Per our results regarding the *instructional support* domain, interaction quality was observed in the upper-middle range. Aligned with the CLASS-S, the results indicated that work tasks were generally clearly presented and that strategies were used to facilitate students’ higher-order thinking, metacognition, and deeper understanding (36). However, within this domain, we observed the lowest interaction quality in the *analysis and inquiry* dimension, which represents cognitive and metacognitive strategies that stimulate students’ engagement in problem-solving (36). In previous studies, nursing and medical students have reported that they derive greater educational value when supervisors challenge their knowledge by asking questions and creating space for students’ reflections, thus contributing to developing deeper knowledge, confidence, and independence (12, 21). Skaalvik et al. (26) reported that when supervisors fail to engage in professional reflections, nursing students perceive missing opportunities for knowledge development. In Giske et al. (60), medical students reported developing independent thinking skills if the supervisor facilitated student reflection before providing an answer to the challenge in question. Based on our results, nursing and medical students in municipal emergency care units had learning opportunities to reflect on patient situations and ethical dilemmas. Notably, we found middle-quality interactions, which, however, suggest possibilities for improvement; supervisors can increase their awareness regarding facilitating students’ higher-order thinking skills. Overall, the CLASS-S tool was found useful to map interaction quality related to possibilities for students’ reflections and development of knowledge.

## Limitations

While the CLASS-S was developed to examine the quality of teacher–student interactions in secondary and upper secondary schools (36, 37), we used it to map the overall interaction qualities for nursing and medical students in primary care placement in two municipal emergency care units. Using the CLASS-S formed a novel approach to study interaction qualities with a new population and setting, for which the CLASS-S has not been validated. Thus, it became necessary to assess face and content validity (34, 61). To assess validity, several measures were carried out. The authors who were to systematically observe nursing and medical students, carefully studied the CLASS-S manual and Secondary CLASS Score Sheet to understand the background, content, and how quality of interactions could be scored (36, 43). In addition, the second author who is a certified user of the CLASS-S, instructed in detail about the CLASS-S domains, dimensions, procedure for observation, and scoring. The second author is a professor of pedagogy, and the third author is a professor of nursing science and pedagogy. They provided professional insight to assess whether the underlying pedagogical basis for the CLASS-S could also be suitable for adult students such as nursing and medical students, which all authors agreed.

Because the CLASS-S is intended to reflect the resources available to students and interaction qualities can be observed in groups with several teachers and students (36), we considered that the CLASS-S dimensions and overall measure could be used to study nursing and medical students’ overall quality of interactions with nurses, physicians, peers, and patients in relevant CoPs (15). However, instead of focusing on the supervisor (36), we focused on either a nursing or medical student when coding interaction behavior. In addition to observing interaction qualities in groups where students, supervisors and/or patients participated, we were open to observe interaction qualities in groups with two participants (e.g., student–student or student-patient). In this context, we realized that a supervisor would be missing, but that it might still be possible to code interaction qualities based on the CLASS-S. Based on thorough review and discussions of the CLASS-S intention, content, and planning how to adjust and use this analysis tool, we considered that the CLASS-S can be useful for studying interaction qualities in municipal emergency care unit placement for nursing and medical students and provide valid results. However, we recognize that the CLASS-S should be further tested in clinical settings for nursing and medical students to strengthen evidence of validity.

In this study, we used Cronbach’s alpha to determine the internal consistency reliability and found that the alpha values ranged between low and acceptable levels (54). In the *emotional support* domain, the alpha values were low and in the domain *framework for learning* the values were low for both professions and the nursing students, and acceptable for the medical students (Table 3). However, previous studies which have used the CLASS-S in school settings, have reported acceptable alpha values in the above-mentioned domain levels. For example, Virtanen et al. (41) calculated alpha values for *emotional support* at 0.83, and *organizational support* at 0.82. Gitomer et al. (62) calculated alpha values for *emotional support* at 0.83, and *classroom organization* at 0.75.

The low alpha values in this study can be attributed to the fact that these domains contain two and three dimensions, respectively (54, 63). Thus, reporting the mean inter-item correlation values can be a better measure of internal consistency (54). Briggs and Cheek (64) argue that the optimal mean inter-item correlation values range between 0.2 and 0.4, and if higher than 0.5, the items may be redundant. In this study, the mean inter-item correlations in the emotional support domain ranged between 0.21 and 0.48, which is acceptable, and in the framework for learning domain between 0.19 and 0.51, which is also acceptable. Further, the low alpha values in this study can be explained by the professional setting in higher education that occurs during clinical placement in municipal emergency care units for nursing and medical students. Within the emotional support domain, we observed that there was a polite and respectful atmosphere where the participants were aware of each other and where positive effects such as smiles and laughter were often unnatural. In the framework for learning domain, we observed that students met behavioral expectations and largely knew what they were supposed to do, and participated actively in learning situations, which may explain the low degree of negative climate, which is good. Negative climate should be low since it might be a hinder for quality work (36).

Although the domains emotional support and framework for learning showed low alpha values, the mean inter-item correlations showed acceptable values. We nevertheless suggest that these domains should be considered related to be used as they are in further studies in municipal emergency care units or similar settings. However, it is interesting that the framework for learning domain showed acceptable alpha and mean inter-item values for the medical students. In the instructional support domain, the alpha and mean inter-item values were within acceptable levels. This domain focus on learning activities with the aim of delivering quality in what the students are supposed to learn or do. The high alpha values indicate that this domain has a high degree of internal consistency and can be used in further studies in the same or similar settings.

While this study’s results cannot be generalized (34), they may reflect interaction qualities for nursing and medical students in primary care placement in the municipal emergency care units. The results, based on the CLASS-S, may be used to develop clinical placement arenas and supervisors’ awareness of interaction quality to support student learning. Furthermore, the results may enhance the knowledge regarding interaction quality in primary care placement, which can be transferable to nursing and medical education in all clinical placement arenas and among supervisors, students, and others involved in students learning.

The systematic observations in the cycles (*n* = 201) ranged from 6 to 71 min; thus, some did not align with the CLASS-S manual (36). As the observations were conducted in live clinical settings with real patients, a precise determination of when to start and stop the observation cycle was challenging. However, Vattøy and Gamlem (65) demonstrated that shorter and longer observation times within the cycles (5–15 min) can capture aspects of teacher–student interactions. Accordingly, we considered that shorter and longer observation cycles could adequately describe interaction quality. The large number of observation cycles applied herein strengthened this study’s validity. To avoid compromising validity, we decided that all observation cycles would have a minimum observation time of 6 min.

## Conclusion

This study aimed to systematically map interaction quality for nursing and medical students in primary care placements in two municipal emergency care units. Based on the CLASS-S, the interaction quality indicated a generally positive and supportive learning environment contributing to students’ learning and active participation in work tasks related to their professional roles. Thus, this new form for primary care placement in the municipal emergency care units for nursing and medical students was found to be a positive learning arena. The results may enhance nursing and medical education programs in countries with similar health services and education. Health education, supervisors, peers, and others contributing to students’ learning, should recognize which interaction qualities may affect learning and how to improve the quality, thus affecting supervisors’ approach to training students. While the CLASS-S showed potential for mapping interaction quality for nursing and medical students in primary care placement in municipal emergency care units, further studies are needed to pilot and validate the CLASS-S for use in clinical placement settings.

## Data availability statement

The raw data supporting the conclusions of this article will be made available by the authors, without undue reservation.

## Author contributions

SG, SMG, MK, BJL, TH, and BMD contributed to the conceptualization and methodology of this study, carefully reviewed and edited the manuscript. SG, MK, BJL, and BMD collected the data. SG, SMG, and TH analyzed the data. SG wrote the first draft of the manuscript. All authors approved the final version of this manuscript.

## Footnotes

^1^The Norwegian Directorate of Health: https://helsedirektoratet.no/Lists/Publikasjoner/Attachments/133/Kommunenes-plikt-til-oyeblikkelig-hjelp-dognopphold-veiledningsmateriell.pdf

^2^ https://explorable.com/descriptive-research-design

^3^ https://store.teachstone.com/score-sheets/

^4^ https://store.teachstone.com/score-sheets/
